# Biomimetic gradient hydrogels for osteochondral regeneration: from multi-dimensional design to clinical translation

**DOI:** 10.3389/fbioe.2026.1860613

**Published:** 2026-07-03

**Authors:** Dohee Kim, Seihyun Park, Seunghun S. Lee

**Affiliations:** Department of Biomedical Engineering, Dongguk University, Seoul, Republic of Korea

**Keywords:** 3D bioprinting, biomimetic scaffold design, clinical translation, gradient hydrogels, osteochondral regeneration, tissue engineering

## Abstract

Osteochondral defects represent a significant clinical challenge due to the complex, graded architecture of the osteochondral unit and the limited regenerative capacity of articular cartilage. Gradient hydrogels have emerged as a promising class of biomimetic scaffolds that replicate the continuous transitions in structure, mechanics, composition, and biochemistry spanning from cartilage to subchondral bone. This review comprehensively examines recent advances in gradient hydrogel design for osteochondral regeneration. We discuss fundamental design principles encompassing structural porosity gradients, mechanical stiffness gradients (kPa-to-GPa transitions), compositional mineral gradients, and biochemical growth factor gradients, as well as their multi-dimensional integration. Fabrication strategies—including layer-by-layer assembly, 3D bioprinting, microfluidic generation, diffusion-based methods, and injectable *in situ* systems—are critically compared regarding scalability, precision, and translational potential. Material selection spanning natural polymers (collagen, gelatin methacryloyl (GelMA), hyaluronic acid, silk fibroin), synthetic polymers (polyethylene glycol, polycaprolactone), and polymer-ceramic composites is evaluated alongside crosslinking chemistries enabling spatial control. We examine biological mechanisms through which gradients direct cell fate, including mechanotransduction via Yes-associated protein/transcriptional co-activator with PDZ-binding motif signaling, spatiotemporal growth factor delivery, zone-specific cell migration, immunomodulation, and extracellular vesicle-mediated paracrine signaling. Preclinical evidence from small and large animal models is synthesized, demonstrating superior outcomes for gradient versus uniform scaffolds. Finally, we address current limitations in long-term durability, vascularization, and regulatory pathways, and highlight emerging technologies—4D printing, AI-aided design, organ-on-chip screening, and personalized medicine—that may accelerate clinical translation.

## 1 Introduction

### 1.1 Clinical challenge of osteochondral defects

Osteochondral defects represent one of the most challenging orthopedic problems, affecting millions of patients worldwide. These injuries involve simultaneous damage to articular cartilage and the underlying subchondral bone, commonly resulting from traumatic injury, osteoarthritis, or osteochondritis dissecans (Nooeaid et al., 2012). Articular cartilage lesions are present in 60%–66% of knees undergoing arthroscopy, with full-thickness chondral defects reported in approximately 5%–6% of all arthroscopic examinations (Curl et al., 1997; Hjelle et al., 2002). Large defects (>2 cm^2^) carry particularly poor prognosis due to the avascular, aneural, and alymphatic nature of articular cartilage, which severely limits its intrinsic regenerative capacity (Buckwalter and Mankin, 1998).

Current clinical interventions remain suboptimal. Marrow stimulation techniques such as microfracture provide short-term symptom relief but generate mechanically inferior fibrocartilage rich in type I collagen rather than hyaline cartilage (Mithoefer et al., 2009). Autologous chondrocyte implantation (ACI) requires two-stage surgery, carries donor-site morbidity, and shows variable long-term outcomes, with chondrocytes prone to dedifferentiation during *in vitro* expansion (Brittberg et al., 1994). Osteochondral autografts are limited by donor tissue availability and morbidity, while allografts face immunological rejection risks (Buckwalter and Mankin, 1998). More critically, these therapies generally fail to simultaneously restore the distinct cartilaginous and osseous phases alongside their critical transitional interface. Biphasic scaffolds have shown promise but often suffer from delamination at the interface due to stress concentrations and mechanical mismatch (Schaefer et al., 2000; Martin et al., 2007). These fundamental limitations underscore the urgent need for advanced tissue engineering strategies capable of regenerating the entire osteochondral unit as an integrated structure.

The global burden of osteochondral pathology is substantial. Osteoarthritis (OA), the most common joint disease worldwide, affects over 500 million people globally and is projected to become the fourth leading cause of disability by 2030, with osteochondral damage as a central pathological feature (Leifer et al., 2022). In younger populations, sports-related injuries account for a significant proportion of osteochondral lesions, with up to 36% of athletes’ knees showing chondral defects upon arthroscopic examination. The direct and indirect economic costs—including surgical interventions, rehabilitation, lost productivity, and long-term disability management—account for an estimated 1%–2.5% of the gross national product in countries with established market economies, with average annual per-patient costs ranging from $700 to $15,600 (Leifer et al., 2022). These epidemiological realities emphasize the pressing need for regenerative solutions that can provide durable, functional tissue restoration rather than merely palliative symptom management.

### 1.2 The osteochondral unit: Structure and composition

**FIGURE 1:**
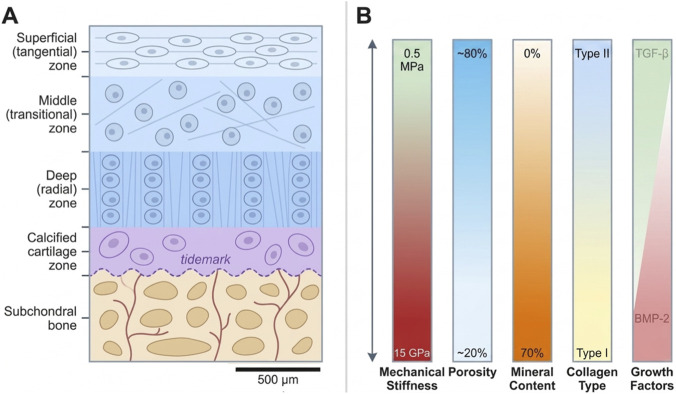
Schematic illustration of the native osteochondral unit showing zonal architecture (superficial, middle, deep, calcified cartilage, subchondral bone) and corresponding multi-dimensional gradients. **(A)** Anatomical zones with characteristic cellularity and collagen orientation. **(B)** Gradient profiles: mechanical properties (stiffness increasing ∼1000-fold from cartilage to bone), porosity (decreasing toward cortical bone), mineralization (increasing toward bone), ECM composition (type II collagen and proteoglycans in cartilage; type I collagen and HA in bone), and growth factor distribution (TGF-β in cartilage, BMPs in bone).

The osteochondral unit is a highly organized, hierarchical tissue exhibiting continuous gradients in cellularity, extracellular matrix (ECM) composition, and mechanical properties (Figure 1). The superficial (tangential) zone comprises 10%–20% of cartilage thickness and contains flattened chondrocytes aligned parallel to the articular surface, with type II collagen fibrils oriented tangentially to resist shear forces. The middle (transitional) zone accounts for 40%–60% of thickness, with spherical chondrocytes in randomly oriented collagen and increased proteoglycan content providing shock absorption. The deep (radial) zone (∼30% thickness) contains columnar chondrocytes arranged perpendicular to the surface with radially oriented collagen facilitating load transmission (Mow et al., 1992; Sophia Fox et al., 2009).

Below cartilage lies the calcified cartilage zone (∼5% thickness), characterized by hypertrophic chondrocytes and mineral-rich matrix anchoring to the tidemark. The subchondral bone comprises trabecular and cortical bone with elastic modulus reaching 5–15 GPa. This zonal architecture creates a remarkable ∼10-fold stiffness gradient, from 0.5–2 MPa in superficial cartilage to the GPa range in bone (Sophia Fox et al., 2009). Compositionally, proteoglycan content increases from ∼15% wet weight in superficial zones to ∼30% in deep zones, while type II collagen makes up 50%–70% of cartilage dry weight with zone-specific orientation. Mineralization occurs discontinuously at the tidemark and throughout calcified cartilage (40%–70% mineral by dry weight) (Madry et al., 2010). This multi-dimensional complexity—involving spatial variations in cellularity, ECM composition, mechanical properties, and mineralization—cannot be adequately replicated by homogeneous or simple biphasic scaffolds.

### 1.3 Rationale for gradient hydrogels

Within this context, hydrogels have garnered immense interest as premier biomaterial candidates for osteochondral tissue engineering. As three-dimensional, highly hydrated polymeric networks, hydrogels uniquely emulate the physicochemical properties of native ECM, with water content often exceeding 90%, inherent biocompatibility, tunable biodegradability, and diffusive transport properties facilitating nutrient and oxygen exchange (Drury and Mooney, 2003; Peppas et al., 2006). However, traditional uniform hydrogels possess relatively weak mechanical strength and lack the spatial cues necessary for dual-tissue regeneration.

Gradient hydrogels address these limitations by incorporating continuous or discretely-graduated transitions in porosity, stiffness, composition, and biochemical signaling within a single construct. Rather than creating sharp interfaces characteristic of biphasic designs, gradients enable smooth mechanical and biological transitions that distribute stress more favorably, reduce interfacial stress concentrations, and prevent the delamination that plagues layered implants (Hutmacher, 2000; Schaefer et al., 2000; Peng et al., 2023). Concurrently, these spatially organized cues actively orchestrate zone-specific regeneration: mechanical gradients drive mechanotransduction pathways directing mesenchymal stem cells (MSCs) toward chondrogenesis in softer, transforming growth factor-β (TGF-β)-rich regions and osteogenesis in stiffer, bone morphogenetic protein-2 (BMP-2) or mineral-rich regions (Vincent et al., 2013; Gao et al., 2019). By synergizing structural biomimicry with directed biological orchestration, gradient hydrogels offer aadvanced approach for seamless osteochondral regeneration.

To ensure clarity throughout this review, it is important to explicitly define the terminology used to describe these spatially organized constructs. We categorize these systems into four distinct structural types: (1) discrete biphasic scaffolds, which feature a sharp, singular interface between two different compositions; (2) multilayer scaffolds, consisting of three or more stacked, but compositionally discrete layers; (3) stepwise gradient scaffolds, which are graded but contain a finite number of distinct transitional zones (e.g., fabricated via layer-by-layer assembly); and (4) continuous gradient scaffolds, characterized by mathematically continuous, smooth transitions in material properties without discernible internal interfaces. These definitions are used consistently throughout this manuscript to accurately categorize the discussed hydrogel systems.

It should be noted, however, that whether continuous gradients fully eliminate delamination *in vivo* remains under debate. Mismatched *in vivo* degradation rates between cartilage- and bone-side regions, insufficient interfacial crosslinking, and cyclic loading-induced micro-cracks at gradient transitions can still produce interfacial failure, even in nominally continuous designs. Comparative biomechanical studies of biphasic versus gradient constructs have therefore yielded mixed evidence, and we discuss this nuance further in Section 7.1.

### 1.4 Scope and organization of this review

This comprehensive review synthesizes the current state-of-the-art in gradient hydrogels for osteochondral regeneration. We organize our discussion around six core themes: (1) design principles encompassing structural, mechanical, compositional, and biochemical gradients; (2) fabrication strategies with critical assessment of scalability and translational potential; (3) material selection across natural, synthetic, and composite systems; (4) biological mechanisms including mechanotransduction and growth factor signaling; (5) preclinical evidence from animal models; and (6) challenges, emerging technologies, and clinical translation pathways. Throughout, we emphasize biomimetic design principles, highlight knowledge gaps, and critically assess which approaches show genuine translational promise toward clinical implementation (Khademhosseini and Langer, 2016).

## 2 Design principles of gradient hydrogels

**FIGURE 2:**
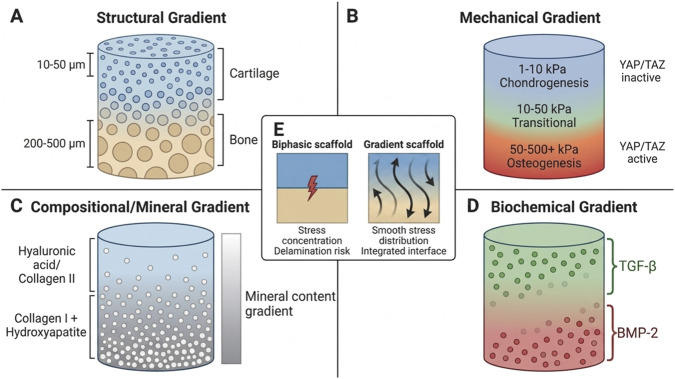
Classification and design principles of gradient hydrogels for osteochondral regeneration. **(A)** Structural gradient showing a continuous pore size transition from small pores (10–50 μm) in the cartilage layer to larger pores (200–500 μm) in the subchondral bone layer. **(B)** Mechanical gradient displaying a stiffness transition from a soft chondrogenic zone (1–10 kPa) to a stiff osteogenic zone (50–500+ kPa), regulating YAP/TAZ activation. **(C)** Compositional/mineral gradient depicting the spatial transition from a Hyaluronic acid/Collagen II phase to a Collagen I and Hydroxyapatite-rich phase. **(D)** Biochemical gradient illustrating the opposing concentration profiles of chondrogenic (TGF-β) and osteogenic (BMP-2) growth factors. **(E)** Mechanical comparison between a discrete biphasic scaffold (exhibiting stress concentration and delamination risks) and a continuous gradient scaffold (facilitating smooth stress distribution and an integrated interface).

The fundamental premise underlying gradient hydrogel design is that the native osteochondral unit functions as a gradient tissue—not a collection of discrete layers—and therefore regenerative scaffolds must incorporate analogous continuous transitions to achieve functional restoration (Figure 2). This biomimetic design philosophy distinguishes gradient hydrogels from earlier-generation monolithic or biphasic scaffolds. Five principal gradient dimensions have been identified as critical design parameters: structural (porosity and pore architecture), mechanical (stiffness and viscoelastic properties), compositional (polymer type and mineral content), biochemical (growth factor and signaling molecule distribution), and temporal (dynamic property evolution during healing). Each dimension targets specific aspects of native tissue complexity, and their integration within a single construct represents the current Frontier of scaffold design (Di Luca et al., 2015; Peng et al., 2023).

### 2.1 Structural and porosity gradients

Structural gradients—variations in pore size, porosity, and interconnectivity—are fundamental design elements influencing cell infiltration, nutrient diffusion, and tissue development. Native osteochondral tissue exhibits structural gradients: cartilage porosity ranges from ∼65% in superficial zones to ∼80% in deeper zones, while bone porosity varies from ∼20% in cortical regions to ∼75–95% in trabecular bone (Hollister, 2005). Pore size gradients ranging from ∼10 to 100 μm in cartilage-mimicking regions to 100–500 μm in bone-mimicking regions direct differential cell infiltration and tissue formation. Smaller pores restrict cell migration and promote chondrocyte establishment; larger pores facilitate osteoblast infiltration and new bone formation with vascularization (Loh and Choong, 2013).

Achieving structural gradients requires precise fabrication control. Porosity can be varied by modulating gelation kinetics, polymer concentration, or crosslinking density in a spatially-controlled manner. Techniques include gradient salt leaching with varying crystal sizes, layer-by-layer salt-particulate incorporation, and 3D printing with variable infill patterns (Yang et al., 2001). Computational modeling has enabled prediction of porosity–permeability relationships, allowing designers to target specific diffusional regimes. However, maintaining structural gradient stability over time remains challenging, as differential polymer degradation or mechanical creep can abolish intended gradients, necessitating careful selection of degradation kinetics matched to tissue regeneration rates.

### 2.2 Mechanical and stiffness gradients

Mechanical gradients are arguably the most critical design dimension, as substrate stiffness directly influences cell phenotype through mechanotransduction. Before detailing these gradients, a brief note on terminology is necessary, as “stiffness” encompasses several distinct mechanical quantities depending on the measurement modality. In this context, quantitative values may refer to Young’s modulus derived from tensile testing, compressive modulus from indentation or unconfined compression, or localized substrate stiffness inferred from atomic force microscopy (AFM) or rheology. Meanwhile, “tissue stiffness” is often used as a broader, context-dependent functional descriptor. Unless otherwise noted, the specific measurement modalities corresponding to the numerical values discussed below have been specified to avoid ambiguity. Native osteochondral tissue exhibits a >1000-fold stiffness gradient: from ∼0.5 MPa in superficial cartilage to ∼8 MPa in the deep zone and 5–15 GPa in subchondral bone (Sophia Fox et al., 2009). Hydrogel stiffness can be tuned over a wide range (1–1000 kPa) through modulation of polymer concentration, crosslinking density, and crosslinker type (Lee et al., 2022; Jo et al., 2026; Lee et al., 2026). However, achieving bone-level stiffness (GPa range) requires incorporation of inorganic phases such as hydroxyapatite (HA) or calcium phosphate (Nukavarapu and Dorcemus, 2013).

The mechanistic basis for stiffness-directed differentiation involves multiple mechanotransduction pathways. *Engler et al.*‘s seminal work demonstrated that MSCs cultured on soft substrates (∼1 kPa) preferentially undergo neurogenic differentiation, intermediate stiffness (∼10 kPa) promotes myogenic differentiation, and stiff substrates (>40 kPa) drive osteogenic commitment (Engler et al., 2006). The YAP/TAZ pathway has emerged as a central mediator: on stiff substrates, YAP/TAZ translocate to the nucleus promoting osteogenic gene expression (RUNX2, osteocalcin), while on soft substrates they remain cytoplasmic, permitting chondrogenic commitment through SOX9 signaling (Panciera et al., 2017) (discussed in detail in Section 5.1). For osteochondral applications, gradient designs typically employ soft interfaces (1–10 kPa) for chondrogenesis, intermediate zones (10–50 kPa), and stiffer regions (50–500+ kPa) for osteogenesis. The gradient slope—the rate of stiffness change over distance—also influences outcomes, though optimal slopes remain poorly defined, representing a key research gap.

Implementing stiffness gradients requires synchronized control of multiple parameters. Spatial variation in crosslinking density can be achieved through UV light intensity gradients, diffusion-limited crosslinking, or time-dependent crosslinking. Incorporation of stiffness-modifying agents (nanoparticles, hydroxyapatite) at gradient concentrations provides another avenue. Sequential assembly of progressively stiffer components offers a third approach. Each method presents trade-offs: mechanical gradients achieved through crosslinking variations may inadvertently create porosity or composition gradients; ceramic incorporation improves stiffness but may reduce hydrogel character and biological activity. *Vincent et al.* demonstrated that stem cells on linear stiffness gradient hydrogels exhibit durotaxis—directed migration toward stiffer regions—suggesting that mechanical gradients can actively recruit and spatially organize cells within constructs, a phenomenon with direct relevance to osteochondral tissue organization (Vincent et al., 2013).

### 2.3 Compositional and mineral gradients

Compositional gradients involve spatial variation in polymer type, blending ratios, or incorporation of bioactive molecules. Mineral gradients—specifically HA or calcium phosphate distribution—are critical for bridging the cartilage–bone interface. Native bone comprises 60%–70% mineral by dry weight; effective synthetic systems typically incorporate 10%–40% mineral content (Madry et al., 2010). Compositional strategies include progressively increasing collagen concentration toward bone regions (promoting osteogenic signaling) and hyaluronic acid toward cartilage regions (promoting chondrogenic signaling).

Several strategies achieve mineralization gradients: (i) *in situ* mineralization using simulated body fluid (SBF) diffusion that deposits minerals preferentially at construct surfaces, (ii) pre-incorporation of mineral at gradient concentrations, (iii) electrochemical mineralization where electric fields guide mineral deposition, and (iv) gradient nanoparticle incorporation within double-network or composite hydrogels, where spatially-graded mineral content creates continuous mineralization transitions mimicking the native calcified cartilage–bone interface (Luo et al., 2015; Fan et al., 2021). *Radhakrishnan et al.* demonstrated an injectable semi-interpenetrating network hydrogel incorporating nanohydroxyapatite (nHA) and chondroitin sulfate nanoparticles in opposing spatial gradients, achieving layer-specific retention of osteoblasts and chondrocytes with complete defect closure in a rabbit osteochondral model. However, excessive mineralization can reduce hydrogel hydrophilicity and biological activity, necessitating careful optimization (Radhakrishnan et al., 2018).

### 2.4 Biochemical and growth factor gradients

Biochemical gradients involve spatiotemporal delivery of signaling molecules to direct zone-specific differentiation. Native osteochondral tissue exhibits growth factor gradients: TGF-β is concentrated in cartilage, particularly in deeper zones, while bone morphogenetic proteins (BMP-2, BMP-9) are enriched in bone matrix (Barry et al., 2001; Chen et al., 2004). Growth factors can be incorporated through: (i) immobilization to specific polymer regions, (ii) encapsulation in biodegradable micro/nanoparticles at gradient concentrations, (iii) controlled release from polymers with regionally-varying degradation rates, and (iv) diffusion-based concentration gradients (Lee et al., 2010).

Dual growth factor gradient strategies—TGF-β concentrated in cartilage regions and BMP-2 in bone regions—more closely mimic native signaling and direct zone-appropriate differentiation (Dormer et al., 2010b; Sun et al., 2020). However, growth factor diffusion across construct regions may create overlapping concentration zones with incompletely understood biological consequences. Temporal aspects of delivery are equally important: most designs employ sustained or burst release, but native tissue likely employs dynamic temporal patterns during healing phases. Optimizing both spatial and temporal delivery profiles remains an active area of investigation (Richardson et al., 2001).

### 2.5 Multi-dimensional gradient integration

The most biomimetic gradient hydrogels integrate multiple gradient dimensions simultaneously. Effective multi-dimensional design requires understanding interactions between gradient types: stiffness gradients should align with structural gradients, compositional gradients should support mechanical gradients, and biochemical gradients should reinforce mechanical signals—creating synergistic rather than conflicting cues (Di Luca et al., 2015; Peng et al., 2023). Computational approaches employing finite element analysis for stress prediction and diffusion modeling for growth factor transport are enabling rational multi-dimensional design rather than purely empirical optimization (Yousefi et al., 2015). More recent data-driven and machine-learning approaches now extend these methods toward multiscale gradient optimization (Guo et al., 2023; Chen et al., 2026).

## 3 Fabrication strategies for gradient hydrogels

### 3.1 Layer-by-layer assembly and sequential crosslinking

**FIGURE 3:**
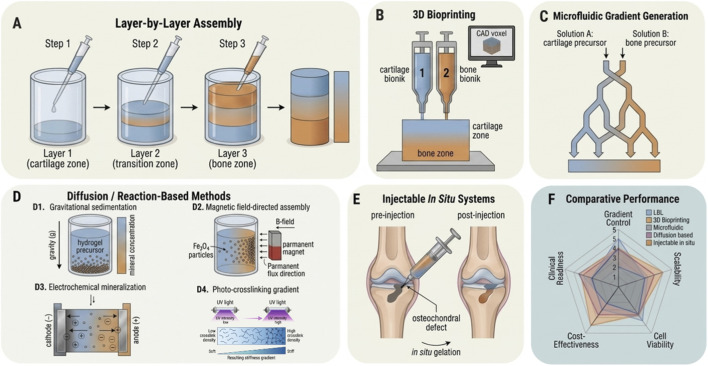
Fabrication strategies for gradient hydrogels. **(A)** Layer-by-layer assembly with sequential casting/coating. **(B)** 3D bioprinting: extrusion-based deposition with variable infill and multi-material capability. **(C)** Microfluidic gradient generation with parallel flow channels. **(D)** Diffusion/reaction-based methods: gradient nanoparticle incorporation, magnetic field-directed assembly, electrochemical mineralization, and light-based crosslinking modulation. **(E)** Injectable *in situ* forming systems with differential gelation. **(F)** Radar charts comparing gradient control precision, scalability, cell viability, and clinical translation readiness.

Layer-by-layer (LbL) assembly involves sequential deposition of gel layers with differing compositions, stiffness, or factor loading, creating discrete approximations of continuous gradients (Figure 3A). The approach offers precise control and excellent reproducibility, making it attractive for clinical translation. Variations include sequential crosslinking with different crosslinkers for each layer, post-deposition modification with bioactive molecules, and intermediate crosslinking to bind layers covalently (Tang et al., 2012). Advantages include straightforward process scaling and capacity to incorporate multiple cell types into appropriate layers. The primary limitation is that discrete layers create stepwise rather than continuous gradients, though thin layer increments can approximate continuity. Interface strength may also be inferior to homogeneous materials. Despite these limitations, LbL assembly remains one of the most clinically-promising approaches due to process simplicity and compatibility with current manufacturing practices.

Recent innovations in LbL assembly include the use of interpenetrating network (IPN) strategies at layer interfaces, where partially-polymerized chains from adjacent layers interdigitate before complete crosslinking, dramatically improving interfacial adhesion and mechanical integration. *Levingstone et al.* demonstrated cell-free multi-layered collagen-based scaffolds with layer-specific regeneration of functional osteochondral tissue in caprine joints, achieving zone-appropriate cartilage and bone tissue formation at 12 months post-implantation (Levingstone et al., 2016). This study represents one of the most compelling demonstrations that LbL gradient scaffolds can direct functional tissue-specific regeneration in clinically-relevant large animal models without exogenous cell delivery.

### 3.2 3D bioprinting and additive manufacturing

3D bioprinting enables computer-aided fabrication of gradient structures with cellular precision (Figure 3B), representing a major methodological advance in scaffold design. Extrusion-based bioprinting deposits cell-laden hydrogels according to digital designs; inkjet bioprinting deposits droplets of different biomaterials sequentially; and laser-based methods (stereolithography, digital light processing) offer higher resolution (Malda et al., 2013; Murphy and Atala, 2014). Gradient implementation includes variable infill density creating structural gradients, spatially-varying crosslinking intensity, sequential printing of different material compositions, and dual-nozzle systems printing multiple materials simultaneously.

Remarkable precision is achievable: layer resolution approaching 50 μm enables feature sizes comparable to cell dimensions. However, cell viability during printing remains challenging—mechanical shear from extrusion can reduce viability to 50%–70%, though systematic optimization has improved this toward >80% (Ozbolat and Hospodiuk, 2016). Material constraints represent a significant limitation: most hydrogels are too viscous for extrusion or too fluid for shape fidelity. Shear-thinning formulations are preferred, where viscosity decreases during printing but recovers post-printing (Highley et al., 2015). *Zhang et al.* demonstrated that 3D-bioprinted anisotropic bicellular living hydrogels could boost osteochondral regeneration in a porcine knee-joint model through reconstruction of the cartilage–bone interface, achieving 23.5% and 20.8% increases in neo-cartilage and neo-bone formation respectively compared with homogeneous hydrogels (Zhang et al., 2024). Scalability for clinical-scale manufacturing remains a challenge, as current systems typically produce constructs <5 cm with printing times of 30 min to several hours.

To illustrate concretely, several recent osteochondral-specific bioprinting studies demonstrate the parameter space currently being explored. *Sun et al.* used dual-nozzle extrusion of GelMA-based bioinks with spatially separated TGF-β3 (cartilage zone) and BMP-4 (bone zone) microsphere depots, achieving zone-appropriate chondrogenic and osteogenic gene expression in a rabbit model at 12 weeks (Sun et al., 2020). *Wu et al.* developed a heterogeneous bilayer construct using a cartilage-side bioink of sodium alginate–calcium with decellularized cartilage ECM and a bone-side bioink containing hydroxyapatite, achieving continuous interfacial regeneration in a rabbit model (Wu et al., 2024). *Zhang et al.* applied 3D-bioprinted anisotropic bicellular living hydrogels combining chondrocytes and osteoblasts in spatial configuration, demonstrating reconstructed cartilage–bone interface (Zhang et al., 2024). *Pati et al.* used decellularized ECM bioinks tissue-matched to cartilage and bone (Pati et al., 2014). Across these studies, layer resolution typically reaches 50–200 μm, post-print cell viability ranges from 60% to 85%, compressive moduli of printed cartilage layers fall between 50 and 500 kPa, and bone-side layers reinforced with mineral phase reach 5–50 MPa. Common limitations include shear-induced viability loss in extrusion, limited resolution at the cartilage–bone interface (∼100 µm), and the difficulty of reproducing the GPa-scale stiffness of subchondral bone with hydrogel-based bioinks alone.

### 3.3 Microfluidic-based gradient generation

Microfluidic systems generate gradients through controlled laminar flow of different solutions through microscale channels (Figure 3C). Concentration gradient generators create linear or non-linear gradients of solutes, while flow-focusing systems converge multiple streams creating compositional interfaces stabilized through gelation (Dertinger et al., 2001). Advantages include precise gradient control and capacity for real-time tuning. However, critical translational limitations exist: extremely small channel dimensions (10–100 μm) limit cell density; continuous flow operation requires external pumping equipment; and generating thick constructs (>1 mm) with through-channel gradients is extremely challenging as diffusion times increase dramatically. Current applications focus on microscale tissue models for studying cellular responses to gradients rather than clinical scaffold production (Sant et al., 2010).

It is important to note that the great majority of microfluidic gradient-generation demonstrations to date have remained as *in vitro* gradient platforms—chips for studying mechanotransduction, drug screening, or cartilage-on-chip disease modeling (e.g., the cartilage-on-chip OA phenotype model of (Occhetta et al., 2019). Direct fabrication of implantable osteochondral constructs via microfluidic methods remains comparatively rare because of throughput limitations (typical fabrication rates of µL/min restrict construct size to ≤5 mm) and the difficulty of integrating high-mineral bone-side regions into laminar-flow workflows. Microfluidic methods are therefore most useful as mechanistic and screening platforms rather than as stand-alone manufacturing routes for clinical-scale constructs.

### 3.4 Diffusion and reaction-based methods

Diffusion-based gradient generation leverages differential diffusion rates through partially-gelled precursors (Figure 3D). Gradient nanoparticle sedimentation employs density differences: mineral-rich nanoparticle suspensions settle through partially-gelled precursors, producing continuous, smooth mineral gradients (Radhakrishnan et al., 2018). Electrochemical mineralization uses electric fields to direct ion migration and mineral deposition. Magnetic field-based methods apply external fields to direct movement of magnetic nanoparticles, creating compositional and mechanical gradients (Ito et al., 2005). *Xu et al.* recently developed continuous mechanical-gradient hydrogels with on-demand distributed Mn^2+^/Mg-doped hydroxyapatite@Fe_3_O_4_, where magnetic field-directed gradient assembly achieved enhanced osteochondral regeneration in rat knee joints (Xu et al., 2025). Light-based crosslinking with spatial light modulation using digital micro-mirror devices can create arbitrary intensity patterns resulting in gradient crosslinking density and corresponding stiffness gradients (Azagarsamy and Anseth, 2013). These methods offer relative simplicity but have limited spatial control and difficulty achieving steep gradients.

Representative osteochondral diffusion-based gradient examples include: *Radhakrishnan et al.*, who developed an in situ-forming nano-engineered composite hydrogel where nanoparticle sedimentation generated a continuous mineral gradient (Radhakrishnan et al., 2018); *Fan et al.*, who fabricated a gradient mineralized and porous double-network hydrogel using simulated body fluid (SBF) immersion to generate apatite gradients that directed BMSC osteochondral differentiation *in vitro* (Fan et al., 2021); and *Wang et al.*, who used dual-gradient silk-based hydrogels for spatially targeted growth-factor delivery and osteochondral regeneration (Wang et al., 2025). Diffusion-based methods produce smooth continuous gradients but require careful control of gelation kinetics: gelation that is too rapid freezes the gradient before equilibration, while gelation that is too slow permits mineral sedimentation beyond the desired profile.

### 3.5 *In Situ* forming and injectable systems

Injectable hydrogels that gel *in vivo* offer distinct advantages for clinical translation (Figure 3E): minimal surgical trauma, arthroscopic delivery potential, and ability to fill irregularly-shaped defects. *In situ* gelation can be triggered by temperature, pH, ionic strength, enzymatic activity, or light (Li et al., 2012). Creating gradients in injectable systems presents unique challenges—the entire system must be flowable pre-injection yet form gradients post-delivery. Strategies include differential gelation kinetics where components gel at different rates, temperature-responsive systems with LCST behavior, and injection of multiple streams maintaining compositional heterogeneity (Yu and Ding, 2008). *Wang et al.* developed a dual-gradient silk-based hydrogel driven by an electrical field that transitions from stiff to soft along the anode-to-cathode direction while incorporated growth factors create a parallel gradient for sustained release, demonstrating significant osteochondral regeneration in a rabbit model (Wang et al., 2025).

Innovations in injectable gradient systems include the development of microgel assemblies—collections of microscale hydrogel particles that can be injected as a slurry and then secondarily crosslinked *in situ* to form a macroscale construct. By mixing microgels of different compositions or stiffness in controlled ratios that vary through the injection depth, compositional gradients can be approximated. Granular hydrogels, composed of jammed microgel particles, offer the additional advantage of inherent porosity between particles, facilitating cell infiltration and nutrient transport. These approaches bridge the gap between injectability and structural complexity, though achieving the precision of pre-formed gradient constructs remains challenging. The development of dynamic covalent chemistry-based injectable hydrogels—where reversible covalent bonds enable initial fluidity followed by progressive stiffening—adds temporal gradient capability to injectable systems, mimicking the progressive mechanical maturation of healing tissue (Yu and Ding, 2008; Li et al., 2012).

### 3.6 Comparison of fabrication strategies

The various fabrication approaches present distinct trade-offs guiding method selection based on application requirements (Table 1; Figure 3F). Layer-by-layer assembly excels in scalability and manufacturing compatibility but produces discrete gradients. Bioprinting offers exceptional design freedom but faces scalability challenges. Microfluidic approaches provide unmatched precision for research but limited clinical applicability. Diffusion-based methods elegantly generate continuous gradients with minimal equipment but offer limited spatial control. Injectable systems maximize accessibility while sacrificing gradient sophistication. The future likely involves hybrid approaches combining advantages of multiple methods. Standardization of processes, materials, and evaluation methods would significantly advance the field and enable better cross-study comparison (Peng et al., 2023; Xiong et al., 2024).

**TABLE 1:** Comparative analysis of gradient hydrogel fabrication strategies.

Strategy	Gradient control	Scalability	Cell viability	Clinical readiness	Specific translational barriers
Layer-by-Layer	Discrete/moderate	High	High (>80%)	High	Large-animal evidence, defect-size scalability
3D bioprinting	Excellent	Moderate	Moderate (60%–80%)	Moderate	GMP compatibility, cost, reproducibility
Microfluidic	Excellent	Low	High (>85%)	Low	Defect-size scalability, GMP compatibility
Diffusion-based	Moderate/continuous	High	Moderate (50%–80%)	Moderate	Reproducibility, large-animal evidence
Injectable *In Situ*	Moderate	Very high	Moderate (50%–70%)	Moderate	Sterilization, regulatory complexity

This table evaluates various fabrication strategies based on explicit objective criteria. Gradient control is classified as discrete (stepwise transitions), moderate (≤3 distinguishable zones), or continuous (analytical/computer-defined functions). Scalability is rated from low (throughput limited to ≤5 mm constructs) to high (compatible with GMP biomanufacturing). Cell viability represents literature-aggregated post-fabrication ranges. Clinical readiness reflects research-stage maturity—based on the number of large-animal studies and demonstrated GMP/sterilization compatibility—rather than regulatory approval, as no gradient-hydrogel product is currently approved for clinical use. Additionally, specific translational barriers (e.g., reproducibility, GMP compatibility, defect-size scalability, regulatory complexity) are explicitly outlined for each method.

## 4 Material selection and crosslinking chemistry

### 4.1 Natural polymers

**FIGURE 4:**
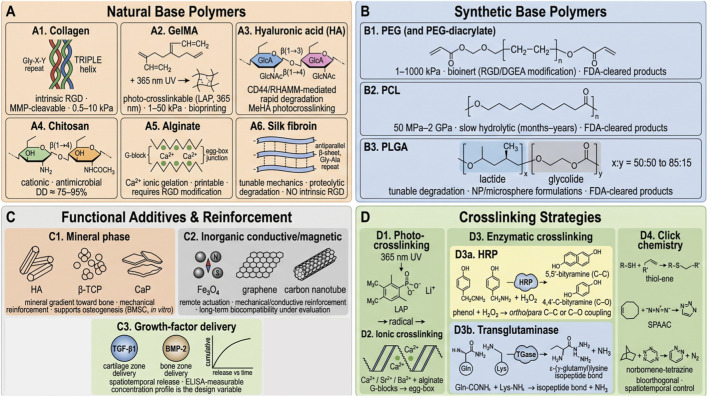
Material toolbox for gradient hydrogels. **(A)** Natural base polymers (collagen, GelMA, HA, chitosan, alginate, and silk fibroin) offering diverse bioactivities. **(B)** Synthetic base polymers (PEG-based, PCL, and PLGA) with tunable mechanical properties and degradation rates. **(C)** Functional additives and reinforcement, including mineral phases for osteogenesis, inorganic nanomaterials for physical or conductive reinforcement, and spatiotemporal growth factor delivery (TGF-β1 and BMP-2). **(D)** Crosslinking strategies, encompassing photo-crosslinking, ionic gelation, enzymatic reactions (HRP and TGase), and bioorthogonal click chemistry.

Natural polymers leverage intrinsic bioactivity and compositional similarity to native ECM (Figure 4). Collagen, the most abundant ECM protein (∼70% of cartilage dry weight), provides RGD sequences for integrin binding and MMP-sensitive sequences for degradation control. Gelatin methacryloyl (GelMA), derived from collagen denaturation with added photo-crosslinkable methacrylate groups, enables rapid UV-induced gelation with tunable crosslinking density and is widely employed in bioprinting due to favorable rheological properties (Nichol et al., 2010; Yue et al., 2015). Gradient stiffness has been achieved in GelMA by varying UV intensity spatially. Hyaluronic acid (HyA), a major cartilage ECM component, provides excellent chondrogenic bioactivity through CD44 and RHAMM receptor interactions. Common derivatives include methacrylated HyA (MeHA) for photo-crosslinking. HyA concentration gradients naturally create biochemical gradients promoting chondrogenesis; however, rapid enzymatic degradation (<2 weeks) limits mechanical stability without modification (Burdick and Prestwich, 2011; Ge et al., 2012).

Chitosan, derived from chitin, provides antimicrobial properties and promotes both chondrogenesis and osteogenesis depending on formulation. Its positive charge enables ionic crosslinking and protein binding (Khor and Lim, 2003). Alginate offers controllable ionic gelation through divalent cations (Ca^2+^, Ba^2+^) and excellent processability for bioprinting and microfluidic applications, though it lacks inherent cell adhesion requiring RGD peptide modification (Lee and Mooney, 2012). Silk fibroin, from silkworm cocoons, offers tunable mechanical properties and β-sheet-mediated cell adhesion. Native *Bombyx mori* silk fibroin heavy chain is dominated by glycine-alanine repeats and does not contain canonical RGD motifs; cell-adhesive performance is typically achieved through β-sheet protein adsorption, surface modification, or recombinant RGD-functionalization rather than intrinsic adhesion sequences (Kundu et al., 2013).

A critical consideration in natural polymer selection is immunogenicity and batch variability. Animal-derived polymers (collagen, gelatin, silk) carry risks of immunological reactions and disease transmission, while plant/microbial-derived alternatives (alginate, bacterial cellulose) avoid these concerns but may contain endotoxins requiring rigorous purification. Recombinant production of collagen and silk proteins is emerging as a solution, offering consistent composition without animal-derived contaminants, though production costs remain high (Chen and Liu, 2016). For gradient hydrogel applications, selecting natural polymers with complementary properties—combining the chondrogenic signaling of HyA with the mechanical stability of silk fibroin or the osteogenic potential of collagen—enables rational design of compositional gradients that synergize with mechanical and biochemical gradient dimensions (Tibbitt and Anseth, 2009).

### 4.2 Synthetic polymers

Synthetic polymers offer excellent control over mechanical properties and degradation kinetics without immunological concerns from animal-derived materials. Polyethylene glycol (PEG) is extensively studied, offering tunable mechanical properties (1–1000 kPa), controllable degradation through incorporated enzymatically degradable peptide sequences, and proven biocompatibility, though it is inherently bioinert requiring peptide incorporation (RGD, DGEA) for cell adhesion (Lutolf and Hubbell, 2005; Zhu, 2010). Poly (ε-caprolactone) (PCL), used in several FDA-cleared medical devices for specific indications, with degradation over months to years and mechanical properties of 50 MPa–2 GPa, is useful for mechanically demanding applications, though its hydrophobicity necessitates blending with hydrophilic polymers (Woodruff and Hutmacher, 2010). Poly (lactic-co-glycolic acid) (PLGA), used in many FDA-cleared products for specific indications, degrades through hydrolysis on tunable timescales and is extensively used in micro/nanoparticle formulations for controlled growth factor delivery, creating compositional and biochemical gradients when distributed at varying concentrations within hydrogels (Makadia and Siegel, 2011).

### 4.3 Composite and hybrid systems

Most successful recent gradient systems use composite approaches. Polymer-ceramic composites incorporating HA or calcium phosphate at gradient concentrations create simultaneous mechanical and compositional gradients. *Xu et al.* employed on-demand distributed Mn^2+^/Mg-doped hydroxyapatite@Fe_3_O_4_ nanoparticles, combining magnetic functionality with osteogenic signals and gradient mechanical properties (Xu et al., 2025). Natural-synthetic polymer blends (e.g., collagen-PEG, HyA-PEG) balance biological activity with mechanical precision (Tibbitt and Anseth, 2009). Nanoparticle-enhanced systems incorporating gold nanoparticles, graphene, or carbon nanotubes at gradient concentrations create mechanical and conductive gradients, though long-term biocompatibility remains incompletely characterized (Shin et al., 2016).

### 4.4 Crosslinking mechanisms

The crosslinking strategy fundamentally determines gradient implementation feasibility. Photo-crosslinking (UV or visible light) offers unmatched spatial precision through digital masks or fiber optics, creating stiffness gradients by modulating light intensity spatially. Advantages include rapid gelation (seconds to minutes) and post-gelation control, though phototoxicity concerns exist with UV systems (Fairbanks et al., 2009; Azagarsamy and Anseth, 2013). Ionic crosslinking with divalent cations is mild and biocompatible; gradient creation occurs through ion diffusion from construct surfaces inward, creating mineral composition gradients. Limitations include weaker mechanical properties and potential instability through ion exchange (Lee and Mooney, 2012). Enzymatic crosslinking (transglutaminase, horseradish peroxidase) offers mild conditions and potential for temporal programming but slower gelation and less precise spatial control (Heck et al., 2013). Dual-crosslinking systems employing two complementary mechanisms—such as photo-crosslinking for rapid mechanical property establishment with enzymatic crosslinking for cell-responsive adaptability—combine rapid processing with biological responsiveness and represent a promising trend (Guo et al., 2019). A complementary, fully bioorthogonal route is strain-promoted azide–alkyne cycloaddition (SPAAC), a copper-free click reaction that proceeds rapidly under cytocompatible conditions without external catalysts or initiators; spatially staged SPAAC and related click chemistries allow gradient crosslink densities to be templated within cell-laden precursors (Figure 4D) (DeForest et al., 2009; Azagarsamy and Anseth, 2013).

### 4.5 Sterilization considerations for material selection

An often-underappreciated constraint in material and crosslinking selection is the requirement for terminal sterilization. Traditional sterilization methods—autoclaving, gamma irradiation, and ethylene oxide (EtO)—can significantly alter the properties of gradient hydrogels. Gamma irradiation at standard doses (25 kGy) induces both crosslinking and chain scission in most polymers, modifying mechanical properties by 10%–50% and potentially eliminating carefully designed stiffness gradients. EtO can leave cytotoxic residues trapped within porous hydrogel networks, while autoclaving denatures protein-based polymers (collagen, gelatin, silk) and degrades thermolabile growth factors. These effects must be considered during the design phase: selecting radiation-stable crosslinking chemistries, avoiding thermolabile components where autoclaving is required, or designing growth factor loading as a post-sterilization step. Emerging sterilization approaches—supercritical CO_2_, low-dose electron beam, and UV-C—show promise for preserving gradient integrity but require validation for each specific formulation to achieve sterility assurance levels (SAL) of 10^–6^ (Pohan et al., 2020).

## 5 Biological mechanisms and cellular responses

### 5.1 Mechanotransduction and stiffness-directed differentiation

Mechanotransduction—the conversion of mechanical signals into biochemical responses—is the foundational mechanism by which mechanical gradients direct cell behavior. The YAP/TAZ pathway is central: Yes-associated protein (YAP) and TAZ are transcriptional co-activators that translocate to the nucleus on stiff substrates, promoting osteogenic gene expression (RUNX2, osteocalcin, alkaline phosphatase), while remaining phosphorylated and cytoplasmic on soft substrates, permitting chondrogenic differentiation. The mechanism involves focal adhesion signaling—cells on stiff substrates develop larger focal adhesions with increased Rho-associated kinase (ROCK) activity, increasing cytoskeletal tension that promotes YAP/TAZ nuclear translocation.

Beyond YAP/TAZ, integrin-mediated signaling through focal adhesion kinase (FAK) and Src kinases transduces mechanical cues. Specific integrin types influence differentiation: α5β1 integrin (fibronectin receptor) promotes osteogenesis; α10β1 (collagen receptor) promotes chondrogenesis (Loeser, 2014). The Wnt/β-catenin pathway, emerging as mechanically-sensitive, shows β-catenin stabilization (osteogenic) on stiff substrates and β-catenin degradation (chondrogenic) on soft substrates (Day et al., 2005). For osteochondral regeneration, soft regions (1–10 kPa) should promote chondrogenesis through suppressed YAP/TAZ, while stiff regions (50–500+ kPa) promote osteogenesis through enhanced YAP/TAZ and Wnt signaling. However, exact stiffness thresholds are cell-type dependent and incompletely defined—most mechanotransduction studies employ MSCs or fibroblasts, with limited data for primary chondrocytes and osteoblasts (Guilak et al., 2009).

A critical nuance often overlooked in gradient hydrogel design is the concept of “mechanical memory”—the observation that cells retain information about past mechanical environments even after transfer to new substrates. *Yang et al.* demonstrated that MSCs cultured on stiff tissue culture polystyrene for extended periods retained nuclear YAP/TAZ and RUNX2 activation even after transfer to soft (2 kPa) PEG hydrogels, with irreversible osteogenic commitment occurring above a threshold mechanical dose (Yang et al., 2014). This suggests that initial mechanical exposure during early scaffold implantation may permanently influence cell fate decisions. This has implications for gradient hydrogel temporal design: if gradients change over time due to degradation or remodeling, cells in transitional zones may either retain their original phenotype or undergo phenotype switching depending on the duration of initial mechanical exposure. Understanding mechanical memory in the context of degrading gradient scaffolds represents an important but largely unaddressed research question.

We note, however, that cell response to substrate mechanics is not determined by linear-elastic stiffness alone. A substantial body of work has highlighted multiple modulating factors that should accompany any gradient design or interpretation: matrix viscoelasticity (stress-relaxation rates can override elastic-modulus effects on differentiation (Chaudhuri et al., 2016), ligand density and tethering (adhesive-ligand spacing on identical-stiffness substrates can yield different differentiation outcomes)(Trappmann et al., 2012), dimensionality (2D vs. 3D mechanotransduction differ qualitatively)(Baker and Chen, 2012), degradability (cells can locally remodel matrix on slow timescales, altering perceived stiffness)(Khetan et al., 2013), and culture conditions including soluble factors and mechanical loading (Yang et al., 2014; Occhetta et al., 2019). We therefore present YAP/TAZ-mediated stiffness sensing as a central but not exclusive mechanism, and recommend that gradient hydrogel design specifications include viscoelastic, ligand, and degradation parameters in addition to bulk stiffness.

### 5.2 Growth factor signaling and spatiotemporal delivery

TGF-β at moderate concentrations (0.1–10 ng/mL) strongly promotes chondrogenesis through SMAD2/3 activation, increasing SOX9 expression and downstream COL2A1 and aggrecan expression (Barry et al., 2001). TGF-β paradoxically can promote endochondral ossification at higher concentrations, highlighting context-dependency. BMP-2 at 50–500 ng/mL promotes osteogenesis through SMAD1/5/8 activation, increasing RUNX2 and Osterix expression (Chen et al., 2004). Mechanotransduction and growth factor signaling converge on common targets: RUNX2 is activated by both stiff substrate signaling (YAP/TAZ) and BMP-2 signaling (SMAD1/5/8), creating synergistic osteogenic effects. Spatial control placing TGF-β in cartilage regions and BMP-2 in bone regions represents an elegant approach, though growth factor diffusion across regions may create overlapping concentration zones with incompletely understood biological consequences (Dormer et al., 2010a; Sun et al., 2020).

### 5.3 Cell migration, homing, and zone-specific differentiation

Structural and compositional gradients promote selective cell infiltration into appropriate zones. Smaller pores (10–50 μm) restrict fibroblast and osteoblast migration while accommodating chondrocytes; larger pores (100–500 μm) facilitate osteoblast infiltration necessary for bone formation with vascularization (Loh and Choong, 2013; Lee et al., 2023a; Lee et al., 2023b). Compositional gradients promote differential cell adhesion through zone-specific integrin-ligand interactions. Cells infiltrating gradient scaffolds often undergo phenotype changes as they migrate into progressively different mechanical and compositional environments—this dynamic phenotype switching, where cells adopt zone-appropriate behavior based on local environment, represents an ideal feature but remains incompletely understood mechanistically (Bian et al., 2013).

An important consideration for osteochondral gradient design is the concept of cell recruitment from endogenous tissue. Cell-free gradient scaffolds that rely entirely on host cell infiltration offer significant advantages over cell-laden approaches: elimination of cell sourcing and expansion requirements, reduced regulatory complexity, lower manufacturing costs, and longer shelf life. Studies have demonstrated that appropriately designed gradient scaffolds can recruit MSCs from bone marrow through the subchondral bone into the scaffold, with mechanical and compositional gradients then directing recruited cells toward zone-appropriate differentiation. Chemokine gradients (SDF-1α, CCL2) can enhance this recruitment when incorporated into scaffold design, though the interplay between chemokine gradients and the mechanical/compositional gradient dimensions is complex and poorly characterized. The success of cell-free approaches depends critically on defect environment—traumatic defects with preserved surrounding tissue show better recruitment than degenerative OA defects with compromised tissue quality (Chu et al., 2010; Levingstone et al., 2016).

### 5.4 Immunomodulation and host response

Immune responses remain significant despite the biocompatibility of hydrogels. Natural polymers generally elicit lower inflammatory responses than synthetic polymers, but degradation products can trigger immune reactions. Ceramic materials are simultaneously immunogenic: calcium phosphate dissolution releases ions affecting immune cell behavior—high calcium concentrations promote M1 (pro-inflammatory) macrophage polarization, while specific phosphate concentrations can promote M2 (anti-inflammatory) polarization (Chen et al., 2016). Incorporating immunomodulatory factors directly into gradients (e.g., IL-10 in cartilage regions, BMP-2 in bone regions) represents an emerging strategy, though most studies to date use gross immunosuppression rather than zone-specific modulation. The foreign body response—chronic inflammatory encapsulation—presents long-term challenges, and gradient complexity may offer unexpected benefits through interfaces that more closely mimic natural tissue transitions (Anderson et al., 2008).

### 5.5 Extracellular vesicles and paracrine signaling

Extracellular vesicles (EVs)—including exosomes (∼100 nm) and microvesicles (100–1000 nm)—are emerging mediators of cell–cell communication in tissue regeneration, carrying proteins, lipids, and nucleic acids that influence recipient cell behavior. Gradient hydrogels enable localized EV production: osteoblasts in bone-mimicking regions secrete osteogenic EVs while chondrocytes in cartilage regions secrete chondrogenic EVs. By spatially organizing cell types, gradients enable zone-specific paracrine signaling reinforcing local differentiation (Tao et al., 2017). *Ding et al.* demonstrated that apoptotic vesicles derived from hypoxia-preconditioned MSCs within modified gelatin hydrogels promoted osteochondral regeneration by enhancing stem cell activity and regulating immunity (Ding et al., 2024). EV capture through heparin or other binding mechanisms is an emerging strategy for maintaining zone-specificity, though this mechanism remains largely unexplored in gradient hydrogel systems.

## 6 Preclinical evidence and *in Vivo* outcomes

### 6.1 Small animal models (rat, rabbit)

**FIGURE 5:**
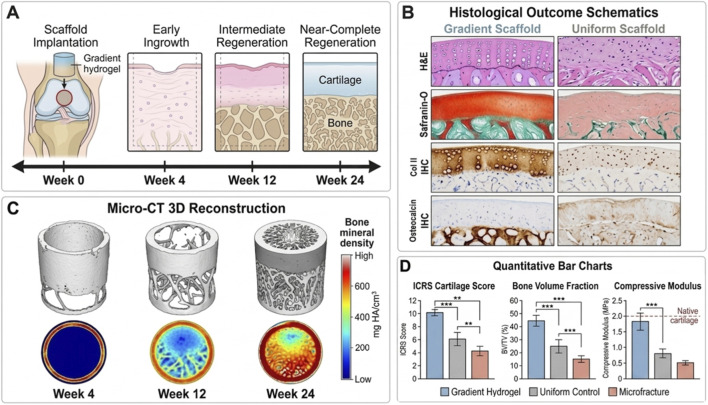
Representative *in vivo* outcomes of gradient hydrogels in osteochondral defect models. **(A)** Schematic surgical timeline at weeks 0, 4, 12, and 24, illustrating scaffold implantation and progressive osteochondral tissue regeneration. **(B)** Histological analysis: H&E (cellularity and organization), Safranin-O (cartilage proteoglycans in red), immunostaining for type II collagen (cartilage) and osteocalcin (bone) showing zone-specific tissue development. **(C)** Micro-CT reconstructions showing bone regeneration progression and mineral density distribution. **(D)** Quantitative analysis: ICRS scores, bone volume fraction, and compressive modulus comparing gradient hydrogel, uniform scaffold, and microfracture control groups; native cartilage compressive modulus shown as a dashed reference line.

Rat and rabbit models provide cost-effective screening platforms with established surgical techniques (Figure 5). Critical-size osteochondral defects are typically 3–5 mm diameter in rats and 8–10 mm in rabbits. Layer-by-layer assembled collagen-based gradients have shown improved cartilage surface integrity and bone regeneration compared to uniform scaffolds at 12 and 24 weeks (Levingstone et al., 2016). *Xu et al.*‘s continuous mechanical-gradient hydrogel with Mn^2+^/MgHA@Fe_3_O_4_ attained remarkable repair effects on full-thickness osteochondral defects in rat knee joints (Xu et al., 2025).

However, small animal models have limitations: rapid healing (8–12 weeks in rabbits vs. >1 year in humans), rapid ossification masking vascularization limitations, small defect sizes inadequately testing scaffold mechanical properties, and different post-operative loading regimens. These factors mean that long-term durability and functional performance remain unassessed in these models (Chu et al., 2010).

A notable recent advance is the development of heterogeneous bilayer hydrogel constructs via dual-nozzle cross-printing with multi-crosslinking inks. *Wu et al.* fabricated a cartilage layer bioink from sodium alginate with calcium ions and decellularized natural cartilage, while the subchondral bone layer consisted of alginate, GelMA, and nano-hydroxyapatite (nHA), creating a bilayer scaffold with gradient crosslinking that achieved stable mechanical properties. *In vitro* biofunctional assessment revealed three orders of magnitude of mRNA upregulation during chondrogenesis and formation of pure hyaline cartilage, while *in vivo* transplantation into rat osteochondral defects demonstrated continuous osteochondral regeneration at 12 weeks (Wu et al., 2024). *Zhang et al.* developed a multileveled hierarchical hydrogel with continuous biophysical and biochemical gradients, synthesizing superparamagnetic HA nanorods (MagHA) that respond to a brief magnetic field to create continuous gradients in nano-hydroxyapatite content, mechanical properties, and magnetism. Applied to rabbit full-thickness osteochondral defects with a local magnetic field, this gradient composite hydrogel achieved repair in a heterogeneous fashion perfectly mimicking the gradual cartilage-to-subchondral bone transition (Zhang et al., 2023).

### 6.2 Large animal models (pig, sheep, goat)

Large animal models (sheep, goat, pig) more closely approximate human anatomy, biomechanics, and healing kinetics. Defect sizes (12–20 mm diameter) better reflect clinically-relevant lesions, and healing extends 6–12 months. A sheep osteochondral defect model evaluating a multilayer gradient collagen/nano-hydroxyapatite composite scaffold demonstrated orderly regeneration with good integration of the chondral surface and significantly improved subchondral bone formation compared to empty defects at 6 months, notably achieving comparable results with cell-free and chondrocyte-seeded constructs (Kon et al., 2010). Goat models evaluating multilayered cryogel scaffolds enriched with chondrocyte-derived exosomes for critical-size osteochondral injuries (8 × 10 mm) demonstrated stratified hyaline cartilage formation with upregulated collagen II, aggrecan, and SOX9 expression at 12 months, with significantly improved subchondral bone regeneration compared to untreated controls (Nikhil et al., 2024). Long-term studies (>6 months) are limited but critical—few extend beyond 24-week observation periods, creating uncertainty about performance on clinically-relevant timescales.

An important gap in large animal literature is the scarcity of head-to-head comparisons between different gradient strategies within the same animal model. Most studies compare a single gradient approach against non-gradient controls, making it impossible to determine which gradient dimensions (mechanical, compositional, biochemical) or fabrication methods provide the greatest therapeutic benefit. Multi-arm studies systematically comparing single-gradient, dual-gradient, and multi-gradient approaches in identical large animal models would substantially advance understanding of which design parameters most critically influence outcomes. Such studies are expensive and logistically challenging but essential for rational clinical development (Chu et al., 2010; Moran et al., 2016). Additionally, functional outcome measures—including gait analysis, joint loading patterns, and long-term pain and function assessment—are rarely reported in preclinical studies but are the outcomes most relevant to clinical translation and patient benefit.

### 6.3 Evaluation methods

Standardization of evaluation methods remains a significant challenge. Histological analysis (H&E, Safranin-O/fast green, immunohistochemistry for type II collagen and osteocalcin) enables semi-quantitative comparison through scoring systems (ICRS, O’Driscoll) (Mainil-Varlet et al., 2003). Micro-CT quantifies mineral density, bone volume, and trabecular architecture in 3D. MRI enables non-destructive assessment of cartilage biochemical composition. Biomechanical testing (unconfined compression, indentation) quantifies tissue mechanical properties, though protocols vary across studies limiting comparability. Molecular analysis via qRT-PCR and RNA-seq assesses zone-specific gene expression (SOX9, RUNX2, COL2A1, COL1A1). Establishment of standardized evaluation protocols would substantially improve cross-study comparison and accelerate field advancement (Moran et al., 2016).

### 6.4 Summary of key *in vivo* findings

A synthesis of recent studies (Table 2) reveals emerging consensus: (i) mechanical gradient importance—gradient-stiffness constructs consistently demonstrate superior regeneration compared to uniform controls through combined mechanotransduction guidance and reduced interfacial stress (Wang et al., 2025; Xu et al., 2025); (ii) multi-dimensional gradient advantage—constructs with simultaneous mechanical, compositional, and biochemical gradients outperform single-dimension gradients, suggesting synergistic effects (Peng et al., 2023); (iii) growth factor spatial control—dual gradient studies show superior dual-tissue development compared to uniform growth factor distribution, though overlap zones show intermediate differentiation (Sun et al., 2020); (iv) injectable system challenges—most injectable systems show inferior mechanical properties compared to pre-formed constructs, with the trade-off between injectability and gradient sophistication remaining a limiting factor (Yu and Ding, 2008); and (v) large defect challenges—while gradient hydrogels perform well for defects <15 mm, larger defects remain problematic due to insufficient nutrient diffusion and mechanical support (Chu et al., 2010).

**TABLE 2:** Summary of representative *in vivo* studies on gradient scaffolds for osteochondral defect repair.

References	Animal model	Defect size	Scaffold type	Gradient strategy	Cell or growth-factor	Reported limitations
Wang et al. (2025)	Rabbit (knee joint)	Ø4mm × 4 mm	Silk-based hydrogel composite	Continuous mechanical gradient	Spatially targeted delivery of bioactive cues	Small animal model; lack of long-term biomechanical evaluation
Xu et al. (2025)	Rat (knee)	Ø1.5 mm × 2.5 mm	Magnetic-responsive composite hydrogel	Continuous mechanical gradient	Mn^2+^/Mg-doped HA@Fe_3_O_4_	Magnetic field reliance for fabrication; potential long-term nanoparticle toxicity
Sun et al. (2020)	Rabbit (knee joint)	Ø4mm × 3 mm	3D-bioprinted polymeric construct	Dual-factor gradient	Dual growth factors (TGF-β3, BMP-4)	*In vivo* degradation-regeneration rate mismatch
Wu et al. (2024)	Rabbit (Osteochondral)	Ø4mm × 4 mm	Heterogeneous 3D-printed hydrogel	Structural pore gradient	Multi-ink cell loading	Confounded mechanical and ceramic contributions
Zhang et al. (2024)	Porcine (knee joint)	Ø4.5 mm × 5 mm	3D-bioprinted hydrogel	Structural pore gradient	Bicellular loading (chondrogenic and osteogenic lineages)	Regulatory hurdles; long-term printed cell viability

This table provides a comprehensive comparison of the core experimental parameters utilized in recent literature. Specifically, it outlines the selection of animal models and the precise dimensions of the induced osteochondral defects, alongside the various scaffold materials and their respective engineered gradient strategies. Furthermore, the table details the integration of biological cues, such as specific cell types or exogenous growth factor loadings. Crucially, it concludes by explicitly identifying the methodological gaps and inherent limitations of each study, thereby offering a critical perspective for future experimental designs.

## 7 Challenges and future perspectives

### 7.1 Current limitations

Despite promising preclinical results, significant challenges limit clinical translation. Long-term durability remains poorly characterized: polymer degradation may eliminate mechanical gradients, and mechanical creep can reduce functional capacity, yet few studies extend beyond 12 months (Chu et al., 2010). Vascularization of larger constructs (>5–10 mm) creates necrotic cores, paradoxically conflicting with the avascular nature of native cartilage. Resolving this requires either temporary vascularization, hypoxia-adapted chondrocytes, or alternative oxygen delivery strategies (Kanczler and Oreffo, 2008). Large defects (>20 mm) remain problematic due to compounding mechanical, diffusional, and manufacturing challenges. Regulatory pathways remain unclear—classification as medical devices, biologics, or combination products requires substantial biocompatibility, sterility (see Section 4.5), and long-term safety data (Huey et al., 2012). Manufacturing costs ($500–5,000 per construct in research settings) must be reduced to $100–300 for clinical adoption, achievable for LbL approaches but challenging for bioprinting. Interfacial strength between gradient regions also requires further optimization, with evidence for superiority over biphasic designs remaining mixed in some studies.

We expand here on three of the most fundamental limitations. First, regarding interfacial integrity and the mixed evidence on delamination superiority: detailed re-examination of the comparative literature suggests that gradient designs reliably outperform discrete biphasic designs only when (i) the polymer chains across the cartilage- and bone-side regions are interpenetrating (IPN strategies), (ii) crosslinking is completed in a single step or with chemically compatible second-step crosslinkers, and (iii) *in vivo* degradation rates of the two regions are tuned to within approximately a factor of two. When *in vivo* degradation rates between regions diverge by more than ∼3-fold, the originally continuous gradient effectively redevelops a sharp interface within weeks, producing the same delamination vulnerability as discrete biphasic constructs. This explains the mixed evidence noted in the literature and identifies degradation-rate matching as a primary design priority. Second, regarding the GPa-scale fatigue loading of weight-bearing joints: pure hydrogels, even with 10%–40% mineral reinforcement, cannot withstand the cyclic GPa-stress regime of native subchondral bone. Realistic clinical paths therefore include (a) restricting initial indications to non- or partial-load-bearing anatomical sites (talar dome, patellofemoral, smaller knee defects), (b) hybrid constructs in which the bone-side region is replaced by a non-hydrogel load-bearing phase (sintered ceramic, PCL/PLGA scaffold, or metal anchor) bonded to a hydrogel cartilage-side region across a graded transition, or (c) staged designs in which the hydrogel scaffold is mechanically protected during early regeneration and is replaced by host-derived bone before peak load resumes. We view (a) and (b) as the most tractable near-term paths; full hydrogel replacement of weight-bearing subchondral bone is, in our assessment, not currently feasible. Third, regarding cost: while LbL approaches can plausibly reach the $100–300 manufacturing-cost range with process automation, custom 3D-bioprinted patient-specific constructs at this price point are unlikely without major advances in printing throughput and standardization.

### 7.2 Emerging technologies

Several emerging technologies offer promise. 4D printing extends 3D bioprinting to include temporal dimensions—constructs designed to change properties over time in response to physiological cues, such as initial stiffness progressively softening as tissue develops (Tibbits, 2014). AI and machine learning algorithms trained on gradient parameter–outcome datasets can propose optimal designs, accelerating iteration beyond human intuition (Guo et al., 2023). Organ-on-chip systems recapitulating osteochondral architecture in miniature permit high-throughput screening of gradient parameters with improved predictive validity (Occhetta et al., 2019). Digital twins integrating computational biomechanics and biology enable patient-specific design optimization. *In situ* monitoring through embedded sensors and responsive scaffolds that adapt to tissue development represent an emerging frontier (Chen et al., 2026). Decellularized ECM (dECM) integration combines the biological complexity of native ECM with synthetic gradient framework advantages, though dECM standardization remains challenging (Pati et al., 2014).

### 7.3 Clinical translation pathway

**FIGURE 6:**
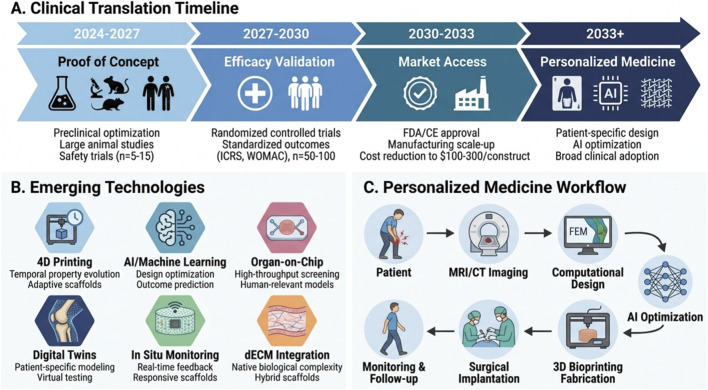
Future perspectives and clinical translation roadmap. **(A)** Timeline from current proof-of-concept through clinical trials to post-market deployment with milestones and enabling technologies. **(B)** Emerging technologies: 4D printing, AI-aided optimization, organ-on-chip screening, digital twins, *in situ* monitoring, and responsive scaffolds. **(C)** Personalized medicine workflow: patient imaging → computational design → personalized fabrication → implantation.

A plausible clinical translation pathway (Figure 6) includes: Phase 1–2 (proof-of-concept, 2024–2027): continued design refinement, large animal study completion, and preliminary human safety/feasibility studies in small cohorts. Phase 2–3 (efficacy validation, 2027–2030): randomized controlled trials comparing gradient hydrogels to standard treatments using standardized outcomes (ICRS assessment, WOMAC scores, structural imaging). Phase 3–4 (optimization and market access, 2030–2033): regulatory approval and commercialization with post-market surveillance. Critical success factors include: demonstration of cost-effective manufacturing at clinical scale, robust regulatory approval pathways, clear superiority in well-powered trials, long-term (5–10 years) safety data, and healthcare provider adoption driven by superior outcomes or cost-effectiveness (Huey et al., 2012).

We emphasize here that the timeline and milestones outlined above are speculative and reflect our authors' assessment rather than demonstrated regulatory pathways. Multiphasic gradient hydrogels carrying growth factors and varying compositions are likely to be classified as Combination Products under FDA guidance (21 CFR 3.2(e)) and equivalent EMA pathways, requiring coordinated review by device, biologic, and drug branches. Establishing batch-to-batch consistency for a continuous gradient is a fundamental QA/QC challenge that, in our view, will require (i) in-process gradient verification (e.g., spatially resolved Raman or near-infrared spectroscopy, micro-CT, or quantitative photoacoustic imaging): performed during or immediately after fabrication on every construct; (ii) statistical process control with explicit acceptance criteria for gradient slope, midpoint, and zone widths; (iii) release-test specifications encompassing viscoelastic mapping, growth-factor content and spatial distribution by ELISA with positional sampling, and sterility; and (iv) a stability program characterizing gradient retention through transport, storage, and clinical preparation. For cell-laden, growth-factor-loaded, and patient-specific bioprinted variants the regulatory burden compounds substantially; acellular and minimally bioactive gradient scaffolds therefore represent the most plausible near-term clinical candidates. Finally, current regulatory pathways treat acellular scaffolds, cell-laden constructs, growth-factor-loaded systems, and patient-specific bioprinted products through different review tracks (e.g., 510(k)/*De Novo*/PMA for devices versus BLA for cell therapies versus combination-product designation), and a sober regulatory strategy must be developed before clinical translation.

### 7.4 Personalized medicine and patient-specific gradients

A compelling emerging direction involves patient-specific gradient hydrogels optimized for individual anatomy, defect characteristics, and biology. This would involve: (i) high-resolution MRI/CT imaging of patient defects with characterization of surrounding bone quality and cartilage properties; (ii) computational design using patient-specific finite element models predicting mechanical stresses under physiological loading and diffusion models predicting nutrient and growth factor transport; (iii) personalized fabrication via 3D bioprinting producing scaffolds matched to patient defect geometry; and (iv) incorporation of patient cells (autologous chondrocytes, MSCs) and patient-derived growth factors (PRP) (Griffith and Naughton, 2002; Lee et al., 2020; Lee et al., 2021).

The convergence of advances in medical imaging, computational modeling, additive manufacturing, and cell therapy makes personalized osteochondral regeneration increasingly plausible within 10–15 years. Early applications might focus on high-impact populations—professional athletes with career-threatening injuries, young patients with large defects who face decades of joint deterioration, or patients who have failed conventional treatments—where the value proposition of personalized approaches justifies higher initial costs. Broader adoption would require cost reduction through manufacturing automation and demonstrated superior outcomes in randomized clinical trials. The integration of real-time intraoperative imaging with on-site 3D bioprinting represents an aspirational but technically feasible endpoint: surgeons could image a defect, design an optimal gradient scaffold, fabricate it chairside, and implant it in a single surgical session. While numerous technical and regulatory hurdles remain, this vision motivates continued dedicated investment in gradient hydrogel research and development (Griffith and Naughton, 2002; Guo et al., 2023).

## 8 Conclusion

Gradient hydrogels represent a substantial methodological advance in osteochondral tissue engineering, moving beyond simple scaffold provision toward biomimetic design that recapitulates the multi-dimensional complexity of native tissue. By systematically incorporating structural, mechanical, compositional, and biochemical gradients, these materials provide an improved capacity to direct zone-specific differentiation and functional tissue development. Fabrication strategies have matured: layer-by-layer assembly offers clinical manufacturing compatibility; 3D bioprinting provides design flexibility and cellular precision; microfluidic methods enable mechanistic understanding; and diffusion-based methods generate elegant continuous gradients. Material selection increasingly employs composite systems balancing natural polymers’ inherent bioactivity with synthetic polymers’ mechanical precision.

Biological mechanisms underlying gradient effectiveness involve synergistic integration of mechanotransduction (YAP/TAZ-dependent differentiation), growth factor signaling (spatial TGF-β/BMP separation), structural guidance (differential cell infiltration), and emerging paracrine signaling through extracellular vesicles. Preclinical evidence consistently demonstrates superior outcomes for gradient versus uniform scaffolds, with multi-dimensional gradients outperforming single-dimension approaches. However, significant translational challenges remain: long-term durability beyond 12 months is poorly characterized; vascularization of large constructs remains inadequately addressed; manufacturing at clinical scale requires process innovation; regulatory pathways need clarity; and cost reduction from research to clinical levels is essential.

Emerging technologies—4D printing, AI-aided design, organ-on-chip screening, digital twins, and personalized medicine approaches—offer compelling pathways to address current limitations. The field should prioritize: (i) completing large animal studies with extended follow-up; (ii) initiating first-in-human clinical trials; (iii) advancing manufacturing automation; (iv) clarifying regulatory pathways; (v) establishing standardized evaluation methods; and (vi) integrating vascularization strategies more systematically into gradient designs. Gradient hydrogels stand at an inflection point between promising research concept and clinical reality. If translational challenges are successfully addressed, these biomimetic materials have genuine potential to transform osteochondral regeneration, moving beyond current palliative treatments toward true functional tissue restoration.

Reframing this synthesis more critically: not all gradient strategies are equally ready for clinical translation. In our assessment, the most realistic near-term clinical candidates are acellular, scalably manufactured gradient scaffolds for small-to-moderate (≤15 mm) non- or partial-load-bearing defects, fabricated by LbL or diffusion-based methods with sterilization-compatible chemistries. Cell-laden, multi-growth-factor-loaded, and patient-specific bioprinted constructs—while scientifically the most exciting—face a much steeper combined regulatory, manufacturing, cost, and reproducibility burden, and are better framed as longer-term opportunities. The weakest assumptions in the field, as we read it, are: (i) that elastic stiffness gradients alone capture the relevant mechanical cue, neglecting viscoelasticity, ligand density, and degradation; (ii) that small-animal short-duration outcomes generalize to human weight-bearing joints; (iii) that continuous gradients automatically eliminate delamination; and (iv) that gradient sophistication scales to clinical manufacturing at a tolerable cost. The most consequential experimental gaps are factorial preclinical studies isolating individual gradient contributions, head-to-head large-animal comparisons of gradient strategies (rather than gradient versus uniform), >12-month durability data, and studies of vascularization in defects above the diffusion-limited size.
